# Historical Origins for the Overestimation of Mammographic Sensitivity

**DOI:** 10.7759/cureus.15940

**Published:** 2021-06-26

**Authors:** Alan Hollingsworth, Abraham N Morse

**Affiliations:** 1 Oncology, Mercy Breast Center, Oklahoma City, USA; 2 Oncology, Auroramri, Boston, USA; 3 Obstetrics and Gynecology, Tufts University Medical Center, Boston, USA

**Keywords:** mammography, screening, breast imaging, sensitivity, breast cancer

## Abstract

The sensitivity of screening mammography for the early detection of breast cancer has improved over the years due to advances in technology. However, guidelines for screening mammography are often based on the mortality reductions demonstrated in the historic trials, where sensitivity with the first-generation mammography was relatively low. With attempts to establish risk:benefit ratios for population screening, it is important to understand the wide range of sensitivities that have been reported for mammography.

Original calculations for mammographic sensitivity were often based on studies that included palpable tumors, thus generating inflated numbers not fully applicable to non-palpable tumors. If restricted to asymptomatic screening, sensitivity calculations were often based on the inverse of interval cancers, a relatively inaccurate method since breast cancers missed on mammography can remain undetected clinically for several years. It was not until multi-modality imaging was developed, primarily ultrasound and MRI, where sensitivity determinations could be made in real time by cross-checking outcomes with each modality. From this, it became apparent that there was a strong correlation between breast density levels and sensitivity levels, such that a single number to denote mammographic sensitivity was disingenuous. The increasing awareness that mortality reductions in the historic trials were achieved with a low sensitivity tool has prompted great interest in additional technologic improvements in mammography, as well as multi-modality imaging approaches for women with high density and/or high risk. In order to appreciate the potential benefit of these new approaches, it is helpful to understand the historical basis behind overestimating the sensitivity of screening mammography.

## Editorial

Since its widespread acceptance in the 1970s and 1980s, mammography has been accorded a high level of sensitivity, often expressed as 90% [1]. This number became so firmly entrenched in the medical community that we still encounter remnants of this belief, such as the disclaimer at the end of some radiology reports that states, “10% of breast cancers are not visible on mammography.”

The misconception of 90% sensitivity (sometimes stretched to 95%) was not without adverse consequences. Women with palpable lumps were sometimes told not to worry in view of negative mammograms, resulting in delayed diagnoses of cancer that would not have occurred in the pre-mammographic era. Soon, there was a malpractice crisis surrounding a common scenario known as Kern’s Triad of Error [2]: 1) young patient, 2) self-discovered mass, and 3) negative mammograms. Eventually, through aggressive educational efforts, a dictum arose: “A dominant mass requires biopsy even if mammograms are negative.”

The origin for the belief in 90% sensitivity is not common knowledge. While one might assume that the historic mammographic screening trials, designed to measure mortality reduction, were the source of the 90% figure, this is not the case. Indeed, during that era, without other forms of breast imaging to cross-check, missed cancers were tabulated through follow-up, usually 12 to 24 months, and sensitivities were low given the crude technology at the time. 

The first randomized trial of mammographic screening intended to reduce cancer deaths was the Health Insurance Plan (HIP) of Greater New York, which began in 1963 [3]. Investigators over the years have attempted to tease out which cancers were discovered by exam, mammography, or both. Eventually, it was determined that mammographic sensitivity for non-palpable cancers in the HIP trial was only 39% [4], a sharp contrast to the 90% figure that would eventually dominate belief.

From the historical screening trials that followed the HIP study, mammographic sensitivity is commonly quoted as a range, e.g., from 71% to 98% [5]. However, a closer look at those trials will reveal that these are the numbers generated from the first screen only, that is, the prevalence screen where tumors are larger. From the clinical standpoint, this can be misleading as we do not base long-term benefits or harms on a single screen. Long-term evaluation of screening mammography is based heavily on the incidence screens where reported sensitivities are 15% to 30% lower than the prevalence screen [5].

The impact of clinical exam has been a powerful confounding variable that has resulted in much of the confusion about mammographic sensitivity and benefit. If one includes clinical exam as part of the screening process, as was done in both limbs of the Canadian NBSS [6], then analysis becomes a greater challenge than when studying mammographic impact alone.

A major contributing factor to the confusion about mammographic sensitivity stems from this lack of a clear definition for “screening,” as to whether or not clinical exam was an integral part of the screening process. The definition that emerged, still in use today, is that screen-detected cancer applies exclusively to the asymptomatic patient, specifically, a negative clinical exam [7]. That is, while clinical exam might be an important tool in routine patient care, a palpable lump (or other sign) excludes the use of the term “screen-detected” if cancer is identified as responsible for the finding on clinical exam.

With this background, it becomes easier to understand how the largest mammographic “screening” trial ever performed was not really a screening trial at all. As mortality reduction through screening mammography began to appear internationally in several prospective, randomized trials, the next question was whether or not the female population in the U.S. could undergo regular screening as a matter of routine. 

Unaware of the magnitude or extent of future screening controversies, the breast cancer leadership in the U.S. would have been better off sponsoring a high-quality prospective randomized trial, especially to sort out screening benefits in the 40-49 age group. Instead, anticipating a revolution in early detection, the National Cancer Institute, with the support of the American Cancer Society, joined forces to stage a massive observational study of feasibility, the Breast Cancer Detection Demonstration Project (BCDDP) [8].

From 1977 to 1980, over 280,000 women at 29 sites underwent five screens using both clinical exam and mammography. When results became available, overall sensitivity in the under-50 group was 90%, while the over-50 group was 95%. Those numbers would remain lodged as dogma for decades. Yet, these numbers were grossly misidentified due to the confounding variable of clinical exam. The oft-quoted “90-95%” represented a mixture of pure mammographic screening and palpable cancers. Lost in the enthusiasm for screening was the fact that nearly one-half of the cancers discovered in the BCDDP were palpable and would have been detected without mammography. Indeed, there were more Stage II patients (n=1,375) diagnosed in the BCDDP than Stage 0 and Stage I combined (n=1306). 

Regardless, the incorrect message of 90%-95% sensitivity for asymptomatic mammographic screening was widely adopted, not only in the lay medical communities but also among breast cancer experts as well. It is difficult to appreciate how this could have occurred, but one must remember that “screening” was a relatively new and nebulous concept at the time, energized by the recent success of the Pap smear. A standard response at the time would have been, “If mammography detects 90% of palpable cancers, why should it be any different for non-palpable cancers?”

Belief in the 90% sensitivity was nearly universal, though some investigators were questioning this number in the early 1990s, recognizing that breast density might have an adverse impact, as well as diffuse tumor histology, as encountered with invasive lobular carcinoma [9].

While it might seem that technologic improvements in mammography would be commensurate with better sensitivity, the impact initially was marginal. In spite of the widespread enthusiasm for conversion to digital mammography, the definitive Digital Mammographic Imaging Screening Trial (DMIST) [10] revealed only a slight advantage in favor of digital over film screen, with this improvement limited to those women with dense tissue. DMIST used two different follow-up intervals to determine sensitivity, one at 12 months and one at 15 months. While media coverage focused on the benefit of digital technology in younger women with dense tissue, little mention was made of the fact that digital sensitivity was only 70% overall at the 12-month interval, while film screen had a sensitivity of 66%. Then, when using 15-month follow-up, both modalities registered weak sensitivity at 41%. 

In a later sub-group analysis of DMIST [11] (10 groups using both technologies), none of the 20 calculations reached 90% sensitivity, including women over 65 with non-dense tissue. Only three of twenty calculations even reached 80% sensitivity. Most concerning was the second largest sub-group (n = 7,315), composed of women under the age of 50, premenopausal, with dense tissue, where film screen sensitivity was a dismal 27%. Digital sensitivity in this same group was 59%, easily reaching statistical significance (p=0.0013). Media outlets gushed over the dramatic improvement with digital mammography, failing to recognize that even 59% sensitivity is far from optimal when the intention is to reduce breast cancer mortality.

The first technologic advance in mammography where improved sensitivity could be seen across the board was 3D tomosynthesis, where mammograms can be studied in “slices” that allow the radiologist to penetrate some of the density. Soon after its introduction, 3D tomosynthesis generated reports of substantial improvements in sensitivity expressed in terms of Cancer Detection Rates (CDRs). This is not a direct measurement of absolute sensitivity, but does allow for indirect comparisons.

For example, the Screening with Tomosynthesis or Standard Mammography (STORM) trial [12] from Italy demonstrated a 5.3/1,000 CDR with 2D digital mammography compared to 8.1/1,000 CDR with 3D tomosynthesis, a relative 53% sensitivity improvement (from 5.3 to 8.1). With some studies of 3D tomosynthesis below this CDR level and some studies above, one thing was clear - the benefit was described and promoted by vendors and health care providers in relative terms, as opposed to absolute numbers, or the more difficult direct measurement of sensitivity. 

To the practicing breast radiologist, CDR rates aside, the benefit became very real when examining 2D reconstructions as normal, but then after the 3D “slices,” recognizing an asymmetric density that proves to be invasive cancer. Clearly, 3D met a clinical need, its strength being demonstrated more easily in dense tissue, with pathology usually invasive (in contrast to controversial DCIS). Whereas detection of invasive disease had previously depended largely on at least one surface of the tumor interfacing with low-density adipose tissue, 3D could detect cancers completely buried in the density (through subtle architectural distortion).

The detailed performance characteristics, including direct measurement of sensitivity and mortality reductions, were to be established by a large prospective, randomized trial known as Tomosynthesis Mammographic Imaging Screening Trial (TMIST) [13]. With multiple endpoints, the study was planned as the most comprehensive breast cancer screening study in decades, enrolling 165,000 women by the end of 2020, randomizing participants to either 2D or 3D tomosynthesis for five years of screening. However, with the early and widespread adoption of 3D technology in the medical community based on evidence available already, accrual to TMIST has fallen well behind targeted enrollment. Facing huge costs in this $100 million trial, it is unclear at the time of this writing whether or not TMIST will be continued [14]. 

Many questions will remain unanswered, and misconceptions will persist if the TMIST trial is discontinued. However, one misconception about 3D has already emerged - a new version of the original overestimation of 90%-95% sensitivity with mammography. Aggressive 3D tomosynthesis promotion has generated the belief that adjunct breast imaging is no longer required for high-risk or high-density patients. However, despite the clear-cut advantages offered by 3D, the improvement in sensitivity, i.e., CDRs, still falls below what is accomplished with adjunct imaging, either ultrasound or MRI.

Much of the ultrasound screening data is grounded in clinical studies limited to women with dense breast tissue on mammography [15], so one must be cautious about extrapolating to general population screening. Still, one can draw these conclusions from available evidence, as applied to women with dense breasts - 1) screening ultrasound will identify more invasive cancers than 3D mammography, and 2) breast MRI will identify more invasive cancers than ultrasound and mammography combined.

Tumor size remains a strong prognosticator in patients with invasive breast cancer. And while tumor biology is emerging as a greater predictor of therapeutic outcome than size, we have little control over biology during the preclinical phase. In contrast, we do have control over tumor size at the time of detection, and this depends on the methodology used for screening. One criticism of dual-modality screening has been that there is no need to identify breast cancer in the sub-centimeter size range, that is, we don’t need to find cancer “earlier” than what we are already doing.

In the case of ultrasound, we are not changing the threshold of detection, that is, mean tumor sizes in studies of ultrasound-detected cancers are generally comparable to mean sizes detected by mammography [16]. When other parameters of ultrasound-detected tumors are analyzed, such as node positivity rates and tumor grade, one can make the claim that ultrasound discoveries should have the same impact on breast cancer mortality reduction as mammographic discoveries. And since there is a greater proportion of invasive cancers when discovered by ultrasound (fewer DCIS cases), one could theorize that, head-to-head, in a population of women with dense breasts, the benefit of screening with ultrasound alone might be preferable to mammographic screening alone.

Although mortality reduction was not an endpoint in the American College of Radiology Imaging Network (ACRIN) 6666 Trial [17], mammographic screening was compared to ultrasound screening in women with dense breasts plus one additional risk factor. Mean tumor size with mammography was 1.15 cm while ultrasound tumors had a mean diameter of 1.0 cm. During the incidence screens (screens 2 & 3), mammographic sensitivity was 52%, while ultrasound sensitivity was 45%. Even using both modalities, combined sensitivity was only 76%. These low sensitivities were tabulated without breast MRI impact, to be discussed below.

ACRIN 6666 took place prior to the introduction of 3D tomosynthesis, so the question arises as to whether or not 3D technology diminishes the need for adjunct ultrasound. To date, the most informative trial addressing this question is the Adjunct Screening with Tomosynthesis or Ultrasound in Women with Mammography-negative Dense Breasts (ASTOUND-2) [18], where each participant had both 3D tomosynthesis and ultrasound added to their negative mammograms. The CDR for 3D tomosynthesis was 2.8/1,000 while ultrasound CDR was 4.9/1,000. Importantly, 27 of the 29 cancers discovered in this trial were invasive, minimizing the risk of overdiagnosis that is more likely with DCIS. 12 of 29 participants had their cancers detected on both modalities, only three were detected with 3D alone, while 14 were detected on ultrasound alone. Thus, if only 3D tomosynthesis had been added to standard mammography, 15 (12 + 3) cancers would have been discovered, with the remaining 14 cancers missed. However, the addition of ultrasound to standard digital mammography yielded 26 cancers (12+14), allowing only three cancers to be missed. 

From a practical standpoint, it’s not an either/or situation - if patients are getting 3D tomosynthesis as their initial screening tool, the addition of screening ultrasound improves sensitivity substantially. The point here is that the benefit of 3D tomosynthesis, albeit well-documented, is currently being overstated, similar to what occurred with the introduction of film screen mammography 40 years ago.

Of course, there are many other issues to consider when extending the screening process to two modalities, including cost, access, the impact of false-positives, etc. However, these factors associated with dual-modality imaging need to be weighed in light of a realistic benefit of 3D tomosynthesis in women with dense breast tissue. So far, this 3D benefit appears to be a long way from improving the sensitivity of cancer detection to a level where an additional modality for high-risk or high-density patients becomes unnecessary.

When MRI is considered as the secondary form of breast screening for high-risk or high-density patients, it must be considered that some of the MRI-discovered cancers are the result of a lower threshold of detection. These sub-centimeter tumors then become the fodder for criticism that we don’t need to find breast cancer “earlier” than mammography. And while this might be true for some of the MRI-detected cancers, if one examines the pathology of MRI discoveries, it is clear that the majority are indistinguishable from those cancers discovered on mammography or ultrasound. So, in the main, while MRI does find some cancers “earlier,” its greater benefit is finding those cancers large enough to be seen on mammography or ultrasound, yet missed with these two modalities.

To that end, returning to the ACRIN 6666 trial [17], the protocol allowed the option of a single MRI screening study at the conclusion of three screening sessions over 24 months using digital mammography and ultrasound. Only one-fourth of participants accepted the offer prior to participation, creating a sub-group of 612 women in whom seven cancers were then identified with mammography and/or ultrasound over the two-year period. 

Remarkably, after what should have been clearance of occult disease through aggressive screening for two years, the single MRI at study conclusion identified nine more cancers, eight of nine being invasive, all node-negative. Mean tumor size was 0.85 cm, smaller than mean tumor size for mammography, yet more likely to have been clinically significant in light of invasion. Whereas the entire original cohort, using both mammography and ultrasound, had a relatively low sensitivity (76%), this dropped even further to 44% once MRI was included, becoming the referent with 100% sensitivity. As for mammograms alone in this study, when compared to MRI, sensitivity was 31.3%, a long way from our starting point of “90%-95%.”

If these low numbers for sensitivity seem improbably low, they are consistent with the international MRI screening studies where, in comparison to MRI, overall sensitivity of mammography in a combined analysis was 40%, and in those studies that included screening ultrasound, sensitivity was still only 43% [19]. This low mammographic sensitivity in women at high-risk or high-density is also consistent with other contrast-enhanced modalities, be it molecular imaging, positron emission mammography, or contrast-enhanced mammography.

Only recently has awareness of lower sensitivity numbers for mammography been accepted. Today, it is common to hear speakers at the podium remind the audience that mammographic sensitivity is “around 80%, though only 50% in women with dense breasts,” numbers that are more easily defended in light of 3D technology. However, this claim makes the dense breast population sound like an outlier. In fact, if we define dense breasts as both Level C and D combined, then nearly half the adult female population fall into the 50% sensitivity group, an unfortunate reality.

This transition to a more realistic appraisal of mammographic sensitivity is necessary in order to justify research into improvements in early detection. If mammograms truly had 90% sensitivity, there was never a need for improvement. However, a small group of researchers, some dedicating their entire careers to breast density, recognized the inflated sensitivity numbers and carried on their efforts to improve early detection. 

Today, screening is under fire as never before, critics arguing that the biology of breast cancer does not allow much benefit through early detection, with overdiagnosis giving the illusion of benefit. But there is an alternative explanation as to why the historic mortality reduction with mammography was marginal - 30 to 40 years ago (there are no modern mammography studies for mortality reduction analysis) radiologists were only finding half of detectable cancers on the incidence screens. That is, the sensitivity in those trials using what is now considered grossly obsolete technology can be estimated at 50%. Half of detectable cancers were in fact detected, but the other half remained in place until large enough to be palpated, or to be detected on the next mammogram one or two years away. So, if finding only half of the “detectable” cancers generates a modest mortality reduction, imagine the impact on mortality if we could find the other half. 

Finding the other half, however, is predicated on the recognition that 90% is a gross overestimate of mammographic sensitivity. With a more realistic estimate of sensitivity, improvements could come through identifying a population that would be better off with ultrasound screening rather than mammography, or alternatively, leveraging third-party payors for better coverage of ultrasound screening as adjunct to mammography. Another innovative approach is no-contrast MRI coupled to artificial intelligence that identifies those patients where contrast is needed. Yet another avenue would be in the development of a screening blood test that selects patients for adjunct ultrasound or MRI if the mammogram is negative, but blood test positive [20]. 

Regardless of the approach under study, the starting point for screening improvements is the recognition that the sensitivity of mammography has been grossly overstated for decades, and the challenge will be closing the gap that is left after the mammogram has been performed.
